# The use of diet modifications and third-party disability in adult dysphagia: The unforeseen burden of caregivers in an economically developing country

**DOI:** 10.4102/sajcd.v67i1.777

**Published:** 2020-11-25

**Authors:** Kim A. Coutts, Maxine Solomon

**Affiliations:** 1 Department of Speech Pathology and Audiology, Faculty of Humanities, University of the Witwatersrand, Johannesburg, South Africa

**Keywords:** adult dysphagia, third-party disability, caregivers, food insecurity, economically developing context

## Abstract

**Background:**

One of the interventions for dysphagia is diet modifications, involving a variety of resources and consumables. In South Africa, where 49% of the population live below the poverty line, the necessities for it are not always feasible for the patient and their family. This coupled with the responsibility of caring for a loved one with disability can culminate into caregivers experiencing third-party disability (TPD).

**Objective:**

To describe the experiences of TPD of caregivers when implementing dysphagia management strategies at home within an economically developing country context.

**Methods:**

This was a qualitative study using phenomenological principles. Data were collected using a semi-structured self-developed interview tool at three tertiary level public sector hospitals. Seven participants and six caregivers were interviewed. Rigour was obtained through credibility, triangulation, transferability, dependability and confirmability. The data were analysed using a thematic content analysis technique following a top-down approach to coding.

**Results:**

The use of diet modification is an appropriate management strategy if the patients’ access and contextual limitations have been taken into consideration. It was evident that the caregivers had multiple International Classification of Functioning, Disability and Health domains affected, which restricted their daily functioning including activities, participation and environmental and personal factors.

**Conclusion:**

The management of dysphagia needs to be family centred and the caregiver’s role and needs have to be considered by all team members when determining long-term management plans. The specific area of how the caregiver’s quality of life was experienced also required further exploration.

## Introduction

Dysphagia (swallowing disorder) is heterogeneous in nature which can result in a variety of serious medical complications, including but not limited to malnutrition, dehydration, difficulty with pill swallowing, aspiration and aspiration pneumonia (Takizawa, Gemmell, Kenworthy, & Speyer, 2016). Management of dysphagia is multifaceted but predominantly falls under the scope of the speech language pathologist (SLP). Management involves a variety of techniques broadly including non-oral feeding methods such as nasogastric and percutaneous endoscopic gastrostomy (PEG) tubes for the more severe cases and swallowing manoeuvres and/or diet modifications (Groher & Crary, 2006) for the people who are able to tolerate some form of oral feeds.

Diet modifications involve altering the consistency of the food to something tolerable for the patient, so that it can be swallowed safely (O’keeffe, 2018). It broadly involves modifications using various techniques such as cooking food for longer, blending and/or using thickening agents (Speyer, Baijens, Heijnen, & Zwijnenberg, 2010) which require access to electricity, stoves, blenders and thickeners, which can be costly. In South Africa, where approximately half of the population live below the poverty line (49.2%) (StatsSA, 2019), the use of this technique can become increasingly challenging because of resource scarcity and contextual constraints. These include lack of access to running water and electricity as well as kitchen items such as kettles and blenders. This scarcity coupled with the responsibility of needing to manage the dysphagia at home can create tension for both the patients and their caregivers. Together with the interest of ensuring that SLP practice patterns are responsive to the needs of the challenging South African context (Moonsamy, Mupawose, Seedat, Mophosho, & Pillay, 2017), the necessity for this article became increasingly significant to bridge this contextual gap.

This article stemmed from a larger study that had a focus of looking at food insecurity challenges related to adult patients with dysphagia who require the use of diet modifications at home. For the larger study, the caregivers were not the original participants; however, the researchers felt that as this theme of third-party disability (TPD) came across as highly pertinent amongst the caregivers during the data analysis process, it required to be its own article.

## Third-party disability

Third-party disability is a consequence of a person’s impairment and how this affects the daily functioning of significant others (Nund et al., 2014). This concept has been scarcely researched, but there is one study in hearing loss (Scarinci, Worrall, & Hickson, 2009) and aphasia (Grawburg, Howe, Worrall, & Scarinci, 2013), respectively. Only one study explored dysphagia in relation to patients with head and neck cancer (HNC) who were undergoing non-surgical therapy, and the findings will be discussed briefly below. The findings from those TPD studies are important for SLPs; however, in terms of understanding caregiver’s experiences in an economically developing context still remains unexplored.

**FIGURE 1:**
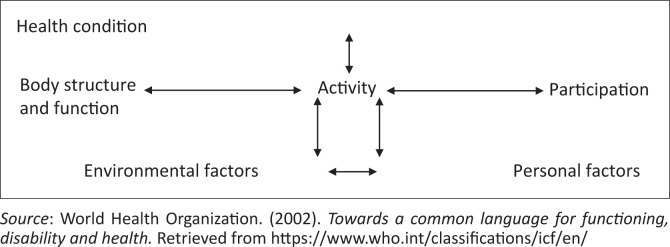
International classification of function and disability framework.

These TPD studies have all utilised the World Health Organization’s framework of the International Classification of Functioning, Disability and Health (ICF) (World Health Organization, 2002) to understand the impact of it on caregivers. This well-known framework is shown in Figure 1.

Threats (2007) used the ICF framework to discuss the impact that dysphagia would have on an individual, using eating as the main activity (in the centre). These challenges include the ability to use utensils appropriately and being able to adequately get the food to the mouth for consumption as a body function. People who have dysphagia because of a neurological deficit may suffer from other comorbidities such as motor impairments, which will further impact on these activities. In terms of participation, Threats (2007) describes these as daily meal preparation as well as actively being able to participate in family and social functions. People with neurogenic dysphagia will need to rely heavily on the assistance of others for both the activity and participatory aspects that surround eating. A challenge with the study of Threats (2007) when needing to relate it to the current article is that the concepts around environmental factors are discussed but focuses solely on the perspective of the person with dysphagia and not the caregivers. Some of the environmental factors that are mentioned include access to support and health care workers (HCWs), as well as their living conditions being adapted to their needs. These findings are helpful but the exact experiences of the caregivers of a person with dysphagia in an economically developing context are yet to be explored.

Researchers who have explored hearing loss and TPD in relation to the ICF found that experiences of caregivers related predominantly to the activities and participation domains followed by contextual factors (Scarinci et al., 2009). In terms of aphasia (Grawberg et al., 2013), these findings were echoed. More to the focus of dysphagia, the article by Nund et al. (2009) that explored TPD in patients with dysphagia post non-surgical treatment of HNC found that the majority of caregiver experiences also correlated to limitations in the activity and participation domains, but there were some hindrances to contextual and personal factors as well. These factors include eating meals at family gatherings as an example. A challenge with these studies is that they have been predominantly conducted in economically developed contexts, such as Australia. Because of a variety of cultural, social, linguistic and economic circumstances of the South African population as well as the complexity of the healthcare system (Coovadia, Jewkes, Barron, Sanders, & Mcintyre, 2009), the specific experiences of caregivers in this context need to be explored to be better understood and incorporated into the context-specific SLP practice teachings, guidelines and policies.

## Methods and materials

This article aimed to describe the caregivers’ experiences in relation to TPD when accessing and fulfilling diet modifications at home. As mentioned, these experiences stemmed from the findings of a larger study that focused on food security challenges in relation to diet modifications at home. The participants for this article are described below in the results section.

This was a qualitative study using phenomenological principles as the researchers were able to gain insights into lived human experiences (Mills & Birks, 2014). Interviews were conducted at three tertiary public hospitals in an urban area. Data were collected as part of the larger study focusing on food insecurity that used a semi-structured self-developed interview tool that was based on themes of the ICF model domains, resource scarcity and diet modification usage. Seven individual interviews were audio-recorded, with each of the participants and their caregivers in a private room when they attended their follow-up appointment at the speech therapy department. Participants for the larger study needed to have been diagnosed with dysphagia and conducting diet modifications at home. There were no specific inclusion or exclusion criteria for the caregivers. The interviews were conducted in English and the duration varied between 10 and 20 min.

## Rigour

For reliability purposes, a pilot study was conducted on one participant to ensure the feasibility and the applicability of the tool and to make any necessary adjustments to the data collection procedure. There were no adjustments relating to the length or content of the tool that needed to be made.

### Credibility

The researchers used member checking and triangulation (Birt, Scott, Cavers, Campbell, & Walter, 2016). The researcher achieved credibility through asking the participants to verify the information that was obtained at the end of the interview (Given, 2015). Environmental triangulation was used as data were collected from three sites (Lichtman, 2011). Theory triangulation was also used as there were multiple perspectives from the literature that were used to review the findings (Lichtman, 2012). Finally, method triangulation was used as data were collected from multiple sources, including patient hospital files, observations and discussions with family members, participants and therapists at the sites (Korstjens & Moster, 2018).

### Transferability

This was ensured by the researcher offering details regarding descriptions of the context, research sites and participant’s information to ensure that the findings were contextually relevant to an economically developing country context (Chilisa & Preece, 2005).

### Dependability

This was obtained by the researcher discussing the findings with her supervisor and other experts in the field. The data from the interviews were transcribed and checked by a research assistant who was not present during the interviews to ensure accuracy (Litchman, 2012).

### Confirmability

The research supervisor assisted with the data analysis and interpretation of the findings (Given, 2015).

### Reflexivity

The main researcher did this through checking her personal biases and keeping a reflective journal throughout the research process (Korstejens & Moser, 2018).

## Data analysis

The data were analysed using a thematic content analysis technique following a top-down approach to coding which involved taking some preset themes from the literature, which in this case was the ICF framework, and then setting up the codes and defining them from the source, which in this case were the interviews (Braun & Clark, 2006). Both authors checked and agreed upon the coding and analysis procedures and findings.

## Results and discussion

### Participant demographics

The demographics of the caregivers and the patients with dysphagia, including their prescribed diet modifications, are described in Table 1.

**TABLE 1:** Participants with dysphagia and caregiver relationships demographics and description of diet modifications.

Participant number	Caregiver–participant relationship	Gender of patient with dysphagia	Race	Age	Cause of dysphagia	Description of solid diet	Description of liquid diet
1	Sister	Female	Black person	33	TBI	Soft	Thickened
2	Mother	Male	Black person	34	Self-extubation (trauma)	PEG: Puree	Regular
3	Husband	Female	Black person	45	MND	PEG: Puree	Regular
4	Husband	Female	Black person	46	MND	Soft	Regular
5	No caregiver	Male	Black person	57	Oesophageal cancer	Soft	Regular
6	Husband	Female	Black person	51	MND	Soft	Regular
7	Wife	Male	Black person	69	MND	Soft	Thickened

MND, motor neuron disease; PEG, percutaneous endoscopic gastrostomy; TBI, traumatic brain injury.

Four (57%) of the seven participants were female with an age range between 33 and 69 years. The mean age for this sample was 47. Four (57%) of the seven participants presented with motor neuron disease (MND). Five participants (71%) were classified as H1 and therefore were receiving some form of income in the family. All (100%) the participants had their solid food intake modified in some manner and two participants (28%) were receiving thickened liquids. Two participants (28%) required PEG feeds. Percutaneous endoscopic gastrostomy feeds are delivered through the tube via a syringe and are therefore required to be of more puree consistency rather than soft, for ease of delivery through the syringe and tube. All participants also reported that they had access to electricity at home. All the caregivers were members of the patient’s immediate family. There was an even distribution between female and male caregivers.

### Objective 1: To describe the methods in which caregivers execute these modified diets at home

To better understand the burden, it was important to describe the type of diet modifications that were being used by the caregivers. Prior to this study, there was little to no available evidence on what caregivers were doing in terms of modifying diets at home, especially in an economically developing country context. Three types of diet modifications used are as follows: two (28%) of the seven participants used thickened liquids, five (71%) of the seven participants used soft food and two (28%) of the seven participants require pureed PEG food. Table 2 describes the different methods that caregivers used to modify the soft food.

**TABLE 2:** Soft diet preparation methods used at home.

Soft food preparation method	Participants (%)	*n*
Eating food that is already soft, for example, porridge	85	6
Overcooking food on stove or oven	100	7
Adding hot water to food	20	1

All the dysphagia participants who ate soft food meant that the caregiver made use of a stove or oven to overcook the food items so that they could be mashed. These participants did report having access to electricity; but in more rural areas, this may be challenging. Participant 1 reported not having access to a blender but used the overcooking vegetables as an alternative. The majority of the participants (85%) reported eating food that was already soft, for example, porridge, custard or *mageu* (a popular sour milk drink in South Africa). This is a convenient option but the considerations from a nutritional perspective for long-term use may need to be possibly investigated.

There were only two participants (28%) who required the use of thickened liquids. Participant 1 used the commercial thickening agent ‘Nutilis’ and the other used a cereal ‘future life’ to achieve the same thickening effect. The cereal is more readily available at retail shops and comes at a much lower cost.

Both the Participants 2 and 3, who used PEG feeds, used blenders to soften the more solid food and they also used naturally soft foods such as *mageu*. For these participants, who live in an urban area and have adequate access to electricity, having access to a blender is of importance for managing this type of diet at home. In the broader South African context where access to electricity (StatsSA, 2019) and specialised kitchen equipment is difficult, this may not always be so easily managed. The use of these types of diet modifications for people who live in more rural areas needs to be considered for future research purposes.

### Objective 2: To describe the impact of third-party disability on the caregivers when executing diet modifications at home

As stated, these findings stemmed from a larger study and are described as they emerged from the data analysis from that larger study. TPD was described as being experienced by all the caregivers (*n* = 6) in the study.

#### Theme 1: The challenges

Five sub-themes emerged in relation to challenges that were identified relating to the use of diet modifications at home: the costs of managing a patient with a disability at home (5/7 participants, 71%), the dislike of the modified diets by the patients (1 participant, 14%), different cultural views related to the use of diet modifications (5/7 participants, 71%), increased dependency (2/7 participants, 28%) and caregiver fear (4/7 participants, 57%).

**Sub-theme 1: Costs:** As the participants who had dysphagia were young and within working age, the caregivers had to bear the burden of being the primary breadwinner and had to run the household with one less income. This would already be a challenge for most households in South Africa, but these families had to bear the extra costs involved in managing a disability with this reduced income.

When using thickening liquids, the caregivers were required to purchase either commercial thickeners at a cost of approximately R200 for a 300 g tin or a cheaper box of non-commercial thickener such as a cereal. The caregiver of Participant 7 stated: ‘I can’t afford but I must try and buy it’ (P6, 70). In a larger urban hospital setting, it is possible that these commercial thickeners are available but because of budget constraints within the healthcare sector, this is not always the case often leaving this cost up to the family. Participant 7 experienced difficulty in accessing these resources from their hospital, whereas Participant 1 was managed at a different hospital and had easier access to these because the hospital had adequate stock available. When caregivers need to supply their own resources, these expenses are a significant contributing factor in their financial decision-making as seen when Participant 2 said: ‘… we are not feeding expensive food’ (P2, 51). Participants 4, 5 and 6 expressed that having to use modified diets was more expensive than before. Participant 1 also stated that the extra costs of medication to manage the main medical diagnosis were another significant expense and that also needs to be taken into consideration over and above the diet.

The indirect costs were described by the caregivers as cost implications around attending multiple hospital appointments and the transport to get to these appointments that were often on different days in conjunction with the need to pay for the essential living costs. Participant 4 reported: ‘I have cancelled some of my appointments’ (P4, 21), as he needed to cancel work appointments to attend required medical appointments with his loved one, thus preventing him from earning an income. This was further described by him as: ‘… causes me to lose out on job opportunities.’ (P4, 21)

Participant 4 reported that living costs are prioritised, for example, rent, and then buying food is secondary as seen in the quote: ‘… we buy food with change’ and ‘… money left is the money we use to buy food and all that’ (P4, 83). It can be confirmed that for these caregivers, the cost of living with a person who has a disability often further exacerbates pre-existing financial difficulties as described by the caregiver of Participant 7 in the quote: ‘I think, can’t afford but I must try and buy it’ (P7, 42).

**Sub-theme 2: Dislike of modified diets by patients:** This was strongly reported by the caregiver of Participant 1, whose sister had suffered a traumatic brain injury (TBI). She was on a soft diet for her solid food intake and also required the use of thickened liquids. The caregiver reported that she: ‘… doesn’t want it …’ (P1, 12) and ‘… yoh, yoh, she complains’ (P1, 15). She further said that she ‘doesn’t finish it’ (P1, 59). Sometimes the caregiver resorted to threatening the patient: ‘… I keep on telling her that we are going back to the hospital’ (P1, 17). As people who have undergone a TBI often display cognitive and pragmatics deficits (Lee et al., 2016), thus having a patient disliking modified foods is a high probability that needs to be considered in SLP management and the impact that this can have on the caregiver needs to be considered.

**Sub-theme 3: Different cultural views on diet modification:** In all, 71% of the participants and their caregivers experienced challenges on differing cultural views regarding the management of their dysphagia. South Africa’s population is diverse with many cultural views of health and illness and how these conditions can be managed (Weber & Kelley, 1998). Participant 3, who has a PEG because of MND, was told by fellow community members that they must have the PEG removed as it was associated with: ‘… evil and bad spirits’ (P3, 56). This creates a significant conflict for both the patient and the caregiver. Participant 1 referred to other food as ‘some are normal’ such as ‘meat’ (P1, 53), implying that the modified diet was not ‘normal’ food as part of their daily meal. Participant 6 refers to the patient having to eat more vegetables, and not meat, unlike the rest of the family.

As South Africa has a diverse population with multiple cultural and religious beliefs, it is inevitable that conflicts in ideologies regarding management will arise. Speech language pathologists need to be more aware of the different beliefs that can impact on the decision-making of the family and patients alike. This is important for holistic and family-centred care (Kenny, 2015).

**Sub-theme 4: Caregiver fear:** This theme of fear emerged strongly from Participants 2 and 3. Participant 3 related her fear to the meal preparation itself and having to feed her loved one via a feeding tube. Her experiences were: ‘… scary and emotional’ (P3, 4). There is a substantial amount of counselling and training that needs to be provided for PEG feeding by the Multidisciplinary team (MDT) (Best & Hitchings, 2010) before discharge to ensure that this is done correctly. Despite this counselling being done, these caregivers still experienced this fear after going home.

The second aspect of fear felt by caregivers was the potential consequences of the dysphagia and the nutritional management at home. The caregiver of Participant 2 stated:

‘I can say it has been difficult. As I have told her that he sometimes vomits and when he vomits, he loses his breaths, he coughs non-stop. So that, that scares me. So yes, it is difficult.’ (P2, 12)

and ‘yeah, it scares us, so it is difficult’ (P2, 24). As mentioned, the medical consequences of dysphagia are significant and as an inexperienced caregiver having to cope with potentially serious complications in an isolated environment clearly creates feelings of justified fear. The caregiver of Participant 1 as mentioned earlier had to use threats occasionally for compliant feeding to be completed. This can lead to a variety of other emotions on the part of the caregiver and the person with dysphagia. Despite this, the comment from caregiver of Participant 1 indicated that: ‘… at the end of the day I have to follow the right instruction’ (P1, 62), despite these real feelings of fear and anxiety that they had to persevere and follow the instructions from the medical staff as they are aware of the consequences.

The implications of this for the MDT are to conduct frequent, appropriate counselling and possible practice sessions prior to the family being discharged. These feelings of fear need to be acknowledged by treating MDT members at follow-up appointments. This led the researchers to question whether the effects of caregiver burden and depression have an impact on compliance during the rehabilitation journey.

**Sub-theme 5: Increased dependency:** The patient of caregiver 4 depends fully on her husband for her care, and this results in him having to: ‘… cancel some of [his] appointments’ (P4, 21) for work, so that medical appointments can be attended. These circumstances were echoed in Participants 2 and 3. Participant 2 needed to be at home as she was the sole person responsible for preparing and administering the PEG feeds. This has had a resultant impact on the sister as she had to move back home to assist with the running of the household. Participant 3 also expressed the need to rely on family members for support. This situation has thus impacted multiple family members with their loss of independence and resulted in a shift of responsibility. Participant 1, who suffered a TBI, had their caregiver report that: ‘… since the accident, I have been at home’ (P1, 41), which has been difficult for her. Participant 1 also reported that: ‘I have to make separate food … it is a challenge’ (P1, 38), showing the significant responsibility in preparing modified diets for a family member. The caregivers expressed that the extra responsibility is something that: ‘It’s part of our life now … so we are getting used to it’ (P1, 70); this shows the resilience and adaptability on the part of the caregivers. In South Africa, family structures and family members’ roles are impacted by culture, which are many. How TPD shifts in responsibilities and increased dependency impacts on these familial structures needs to be further explored.

Based on these findings, it can be said that the caregivers’ TPD affects the following ICF domains: activities, participation and environmental and personal factors. From this study, the activities included the actual act of preparing the meals. Participation included the effect on work-related tasks and how the increased dependency affects family roles and responsibilities. This is currently an under-researched area and a poorly reported topic, especially in terms of caregivers of adult patients with dysphagia. However, the findings from this study do echo some of the findings from the previous studies on TPD that are mentioned earlier by Nund et al. (2009), Grawberg et al. (2013) and Scarinici et al. (2009). The caregivers from these previous studies also had the activities and participation factors of the ICF framework mostly affected. Uniquely, for this study, the environmental and personal factors revealed the repercussions of caregivers’ fear and how differing cultural views can affect the caregiver on a deeply personal level. These findings have not been reported in other studies that have focused on TPD and thus require further exploration.

#### Theme 2: The facilitators

The following facilitators were described by caregivers: appropriate counselling (2/7 participants, 28%), provision of resources from healthcare sites (2/7 participants, 28%), access to state disability grants (4/7 participants, 57%) and family support (100%).

**Sub-theme 1: Appropriate counselling:** Participant 3 expressed gratitude that she was counselled early regarding the progression of MND and how this would have financial implications later on, specifically relating to diet modifications. This counselling was done by the SLP and the dietician. She stated that she ‘had time to get used to it’ as it was ‘scary’ being informed about the need for a PEG. She was counselled about the different types of foods, supplements and specialised equipment that she would need for the PEG feeds. Interestingly, for this participant, she moved from private to public healthcare as she felt that private was too expensive. She mentioned that the resources were more readily available in the public healthcare sector as it supplied her with the necessary consumables, such as the thickening agents. The dietician produced a list of foods that were inexpensive and easily obtainable. In a country where the public healthcare sector has an unpopular reputation, this was a positive finding.

This was similarly reflected by Participant 2 who received counselling from both his SLP and dietician as they were given a list in advance regarding food that would meet their requirements: ‘yeah, none of the list we given is anything expensive or whatever … no … it is something that we can do’ (P2, 39). For patients in a complex setting such as South Africa, adequate pre-counselling is imperative for multiple reasons, including patient compliance and preparation for the rehabilitation journey going forward. There appears to be little published research regarding this, and the current South African policies (South African Speech Language and Hearing Association, 2012) also speak little of this aspect in terms of addressing these issues in SLP scope of practice documents and dysphagia practice guidelines or policies. The hope is that this research can facilitate the start of this change to make our practices more contextually responsive.

**Sub-theme 2: Provisions of consumables from hospitals:** As mentioned earlier, thickeners can be costly. The caregiver of Participant 7 stated ‘[I] can’t afford but I must try and buy it’ (P7, 70). In an urban hospital setting, it is possible that these commercial thickeners are available but because of budget constraints within the healthcare sector, this is not always the case often leaving this cost up to the family. Participant 7 experienced difficulty in accessing these resources from their hospital, whereas Participant 1 was managed at a different hospital and had easier access to these because the hospital had adequate stock available. It can be suggested that hospitals that manage these types of patients need to account for this when developing their annual consumables budget.

**Sub-theme 3: Assistance from state disability grants:** Participant 4, who in the absence of financial support, stated that ‘it is just the grant helping’ (P4, 51), and this helps the family to purchase food. Similar sentiments were echoed by Participants 5 and 6. Participant 7 expressed that ‘the disability grant is helping too much’ (P7, 40), in terms of buying food; however, when it comes to purchasing specialised kitchen equipment, ‘… is still too much’ (P7, 42). These socio-economic factors need to be considered by all team members when doing counselling with patients as well as for management recommendations. Perhaps informing caregivers about disability grants should be an option for inclusion in the pre-counselling process as well as a consideration to referrals to social work to assist with this.

**Sub-theme 4: Family support:** As six of the seven participants had caregivers who were present during the interviews, all of whom were close family members, it can be assumed that family support is imperative to the management of these patients in a home environment.

In terms of the facilitators, the findings from this study are distinctive to the South African context, which is pertinent in terms of being able to translate these results into tangible transformative teaching practices and guidelines.

## Limitations

A significant limitation of this study is that the sample size was small. Because of the structure of the South African healthcare services, the majority of long-term patients are referred down to a primary healthcare level for management or they return home to the rural areas after discharge. Further investigations regarding patients’ experiences at the primary healthcare level and in rural settings are required. This study was conducted in an urban setting consisting of well-resourced tertiary hospitals where family members are often employed. This study needs to be replicated in a rural or semi-rural area as the experiences of caregivers in these settings are expected to be different. As this was also not the primary focus of the larger study, the specific duration of the dysphagia was not fully investigated because this may have an impact on the caregiver as well. This needs to be explored too.

## Conclusion

Dysphagia management is a multifaceted and complex process as it involves not only the rehabilitation of the patient but also a deeper understanding of the role of the caregiver in this social process of eating within their living context. This process also involves multiple healthcare professionals. Diet modification is a useful management strategy for this population; however, access and costs need to be taken into account prior to initiating any management strategy. The concept of TPD in adult dysphagia is complex, but this study made an attempt to understand it by linking it to the ICF framework, as done in previous studies on the topic. The domains of caregivers that are affected are activities, participation and environmental and personal factors. How TPD impacts on the quality of life of the caregivers is also something that needs further investigation. The management of dysphagia needs to be family centred, and the impact of the caregiver’s role needs to be considered by all team members when determining long-term management plans. This study started to shed some light on this important topic and can serve as a springboard for future research and to start transforming current practices to ensure that SLPs become more contextually responsive.
